# MRI coupled with clinically-applicable iron oxide nanoparticles reveals choroid plexus involvement in a murine model of neuroinflammation

**DOI:** 10.1038/s41598-019-46566-1

**Published:** 2019-07-11

**Authors:** Violaine Hubert, Chloé Dumot, Elodie Ong, Camille Amaz, Emmanuelle Canet-Soulas, Fabien Chauveau, Marlène Wiart

**Affiliations:** 1 Univ Lyon, CarMeN laboratory, Inserm U1060, INRA U1397, INSA Lyon, Université Claude Bernard Lyon 1, Lyon, France; 2 HCL, Lyon, France; 3 Clinical Investigation Center, HCL, Louis Pradel Hospital, Lyon, France; 4 BIORAN Team, Lyon Neurosciences Research Center, CNRS UMR5292, Inserm U1028, Université Claude Bernard Lyon 1, Lyon, France; 5 CNRS, Lyon, France

**Keywords:** Neurological models, Magnetic resonance imaging, Imaging the immune system, Translational research, Imaging techniques and agents

## Abstract

Choroid plexus (ChPs) are involved in the early inflammatory response that occurs in many brain disorders. However, the activation of immune cells within the ChPs in response to neuroinflammation is still largely unexplored *in-vivo*. There is therefore a crucial need for developing imaging tool that would allow the non-invasive monitoring of ChP involvement in these diseases. Magnetic resonance imaging (MRI) coupled with superparamagnetic particles of iron oxide (SPIO) is a minimally invasive technique allowing to track phagocytic cells in inflammatory diseases. Our aim was to investigate the potential of ultrasmall SPIO (USPIO)-enhanced MRI to monitor ChP involvement *in-vivo* in a mouse model of neuroinflammation obtained by intraperitoneal administration of lipopolysaccharide. Using high resolution MRI, we identified marked USPIO-related signal drops in the ChPs of animals with neuroinflammation compared to controls. We confirmed these results quantitatively using a 4-points grading system. *Ex-vivo* analysis confirmed USPIO accumulation within the ChP stroma and their uptake by immune cells. We validated the translational potential of our approach using the clinically-applicable USPIO Ferumoxytol. MR imaging of USPIO accumulation within the ChPs may serve as an imaging biomarker to study ChP involvement in neuroinflammatory disorders that could be applied in a straightforward way in clinical practice.

## Introduction

Neuroinflammation is known to be a common factor of many central nervous system (CNS) pathologies, often associated with worsened outcome. CNS inflammatory phenomena need to be thoroughly characterized in order to develop therapeutic strategies aimed at modulating neuroinflammation. Over the years, the status of the CNS as an immune privileged site has been revised, and there are new findings showing that the immune system interacts with the CNS through unique neuroimmunological interfaces^1,2^.

One of these interfaces is increasingly studied in the context of neuroinflammatory disorders: the choroid plexus (ChPs). ChPs are multifunctional organs located inside cerebral ventricles, composed of an epithelial cell layer that surrounds an inner stroma with a network of fenestrated blood capillaries^3^. ChPs are a main component of the blood-cerebro-spinal-fluid-barrier (BCSFB). Thus, they are crucial for immune surveillance of the brain and provide a port of entry for immune cells in a range of neurological diseases^4^. ChPs are involved in the early inflammatory response that occurs after traumatic brain injury^5^, multiple sclerosis (MS)^6^, stroke^7^ and many other CNS pathologies^8^, therefore representing potential therapeutic targets for these disorders^9^. However, the activation of immune cells within the ChPs in response to neuroinflammation is still largely unexplored *in-vivo*. There is therefore a crucial need for developing *in-vivo* imaging tool that would allow the non-invasive monitoring of ChPs involvement in these diseases.

The ChPs are understudied with *in-vivo* imaging, notably because of their small size^10^. The method of reference to image immune cells *in-vivo* in the pre-clinical setting is intravital two-photon microscopy^11^. Unfortunately, the deep localization of ChPs within the cerebrum makes their imaging with two-photon microscope challenging. Furthermore, skull absorption of light prevents clinical translation of this method at this time. Magnetic resonance imaging (MRI) coupled with the intravenous injection of superparamagnetic particles of iron oxides (SPIOs) represents a translational and minimally invasive tool allowing to image immune cells trafficking across the inflamed CNS^12,13^. When injected intravenously, SPIOs are internalized by phagocytic cells, which become magnetic and may thus be detected with MRI. Recently, Millward and coll^14,15^, have shown that this technique allowed to monitor the early involvement of ChPs in a mouse model of multiple sclerosis (experimental auto-immune encephalomyelitis or EAE). These results were obtained using very small SPIOs (VSOP, hydrodynamic diameter range: 6–9 nm) that are similar in terms of physicochemical properties to VSOP C184^14^. VSOP C184 has been tested in human clinical trials up to phase II; however, to the best of our knowledge, it did not receive regulatory approval^16^.

To date, the only commercially-available SPIO that may be administered to patients is Ferumoxytol (Feraheme®, AMAG Pharmaceuticals, United States). Ferumoxytol is an ultrasmall SPIO (USPIO, hydrodynamic diameter range: 20–50 nm) that is Food and Drug Administration (FDA)-approved for the treatment of iron deficiency in adult chronic kidney disease patients and that is being used off-label as an MRI contrast agent^17^. USPIOs have been widely used as imaging probes for tracking phagocytic cells with MRI^18^. In particular, Ferumoxytol-enhanced MRI has been shown to allow the imaging of immune cells trafficking across brain parenchyma in rodent models of multiple sclerosis^19^ and cerebral tumors^20^. However, these works did not specifically examine ChPs involvement. Our aim in the present study was to investigate the potential of USPIO-enhanced MRI to monitor ChPs involvement *in-vivo* in a mouse model of neuroinflammation obtained by intraperitoneal administration of lipopolysaccharide (LPS). Our hypothesis was that active (phagocytic) immune cells present within ChPs would internalize USPIOs and thus become detectable *in-vivo* with MRI. To test our hypothesis, we first used a commercially-available pre-clinical contrast agent, P904 (hydrodynamic diameter range: 25–30 nm), that has the advantage of having a commercially-available fluorescent counterpart, P01240 (Chematech, Dijon, France). To demonstrate the translational potential of our approach, we then replicated the experiments using Ferumoxytol (hydrodynamic diameter range: 17–31 nm).

## Results

**Figure 1:**
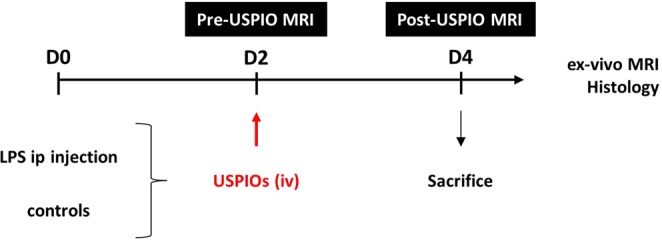
Experimental design. Mice were intraperitoneally (ip) injected with 5 mg of LPS/kg at day 0 (D0). They were imaged by MRI at day 2 (D2) post-LPS administration (pre-USPIO MRI) and then intravenously (iv) injected with USPIOs at the dose of 2 mmol Fe/kg. They were re-imaged with MRI at day 4 (D4) (post-USPIO MRI) and then sacrificed for post-mortem analysis.

Figure 1 shows the experimental design. In brief, neuroinflammation was induced in mice by an intraperitoneal injection of LPS at the dose of 5 mg/kg (unless specified otherwise). This LPS dose has been shown to induce early and prolonged microglia activation and increased expression of brain pro-inflammatory factors, which are maximum one to three days after the LPS challenge^21,22^. A baseline (pre-USPIO) high resolution (7 T) MRI was acquired 48 h post-LPS administration, using a T2-weighted sequence for anatomy and a T2*-weighted sequence to rule out the presence of signal drops before the administration of USPIOs. These sequences were optimized to image the ChPs with sufficient spatial resolution (78-µm in-plane and 500-µm slice thickness) in a period of time compatible with *in-vivo* imaging (total acquisition time: 31 min). Suppl. Fig. S1 shows the location of the different ChPs on MRI: the ChPs of the lateral ventricle anterior horns (LV antH, 3 slices), the ChPs of the third ventricle (3 V, 3 slices), the ChPs of the lateral ventricle body and inferior horns (LV infH, 2 slices) and the ChPs the fourth ventricle (4 V, 3 slices). At baseline, the ChPs may be visible as small structures that are isointense compared to the brain parenchyma and lie inside the hyperintense cerebrospinal fluid (CSF)-filled ventricles. USPIOs were administered intravenously immediately after the baseline MRI. A follow-up MRI exam was obtained 48 h post-USPIOs injection using the same sequences as the baseline exam, the T2*-weighted sequence being optimized to detect USPIO-related signal drops.

**Figure 2:**
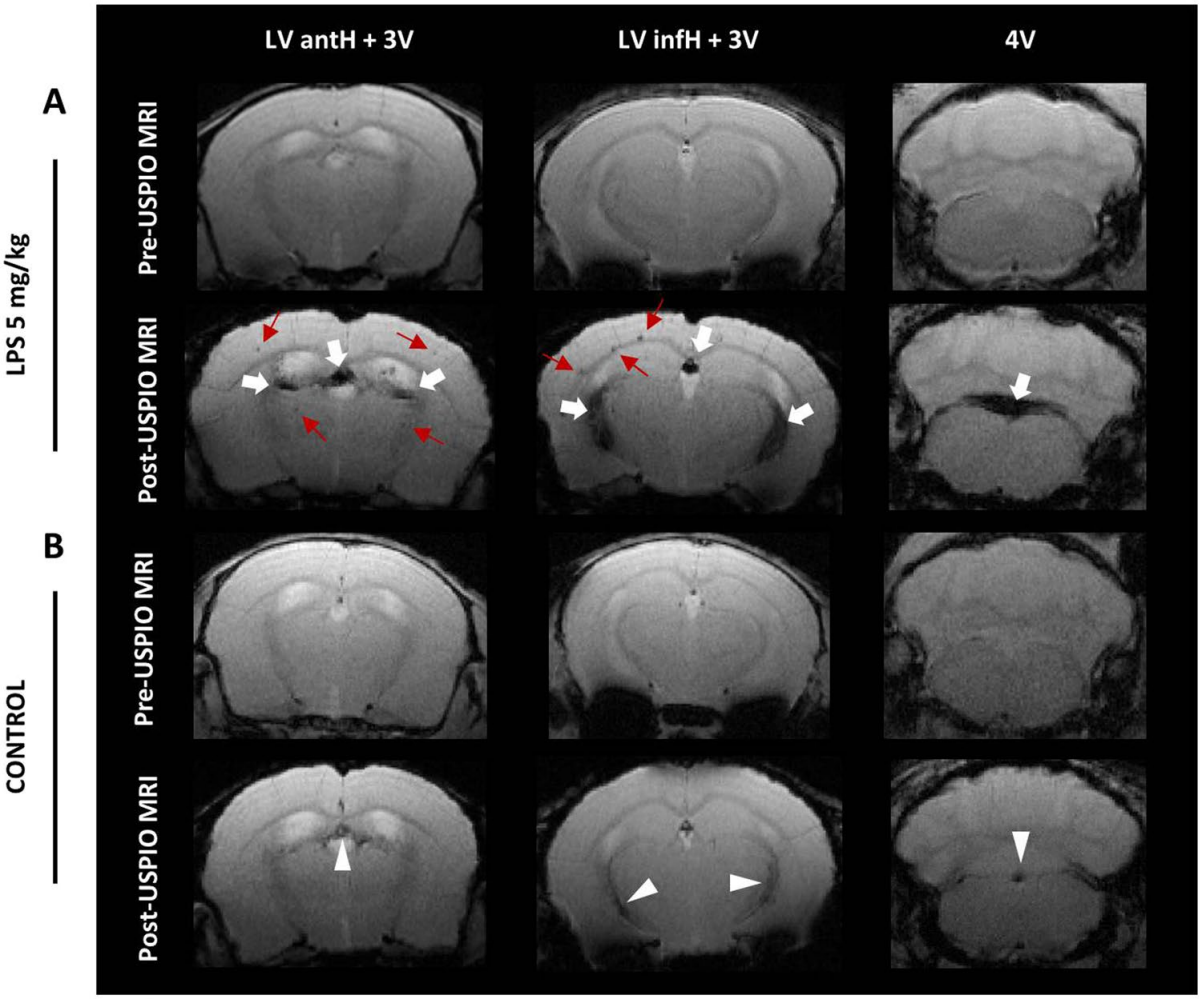
*In-vivo* detection of MR signal drops in ChPs. Pre- and post-USPIO T2*-weighted images of a representative mouse injected with 5 mg LPS/kg (**A**) and a control mouse (**B**), both injected with P904 at the dose of 2 mmol Fe/kg. Only one slice is shown per ChPs location. White arrows indicate marked hypointense signals inside the ChPs of the LPS-treated mouse. Red arrows show hypointense spots inside the brain parenchyma of the LPS-treated mouse. White arrowheads indicate slight hypointense signals inside the ChPs of the control mouse. LV antH: lateral ventricle anterior horns; 3 V: third ventricle; LV infH: lateral ventricle inferior horns; 4 V: fourth ventricle.

First, 15 mice (control, n = 8 and LPS-treated, n = 7) were injected with 2 mmol Fe/kg, either P904 (control, n = 6 and LPS-treated, n = 5) or P904 coupled with rhodamine, i.e. P01240 (control, n = 2 and LPS-treated, n = 2). This dose was chosen to start with because it was shown to be optimal for the MR imaging of phagocytic cells in a mouse model of ischemic stroke^23–25^. Two days post-USPIOs injection, strong hypointense MR signals (MR signal drops) appeared on T2*-weighted imaging in all ChP locations of LPS-treated mice (Fig. 2A, white arrows). Because of the “blooming effect”, the MR signal drops extended beyond the actual ChPs anatomical locations (due to the fact that the local magnetic field created by the USPIOs extends beyond the actual USPIOs location) and made the ChPs readily visible. In addition, several hypointense spots were detected in the brain parenchyma of LPS-treated mice (Fig. 2A, red arrows). In control mice, a few signal drops were also detected within the ChPs when compared to baseline MRI (Fig. 2B, white arrowheads), but they were less marked than in LPS-treated animals. No hypointense spots were visible in the brain parenchyma of control mice.

To evaluate if we could obtain the same results with a lower dose of USPIOs, we injected an additional subgroup of mice (n = 4) with decreasing doses of P904 (1 mmol Fe/kg: LPS-treated, n = 2 and control, n = 1; 0.45 mmol Fe/kg: LPS-treated, n = 1). The marked hypointense MR signals observed in the ChPs of mice injected with 2 mmol Fe/kg were no longer seen with lower doses of USPIOs (Suppl. Fig. S2A). Thus the dose of 2 mmol Fe/kg was used in the rest of the experiments. In turn, to investigate a potential impact of LPS dose on MR signals, another subgroup of mice (n = 6) was submitted to a dose-response study (0 mg LPS/kg, n = 2; 2.5 mg LPS/kg, n = 2; 10 mg LPS/kg, n = 2). One mouse that had received 10 mg LPS/kg died before USPIOs injection. The hypointense MR signals were present in the ChPs of all remaining LPS-treated mice (n = 3), regardless of the LPS dose and with no obvious dose effect (Suppl. Fig. S3).

**Figure 3:**
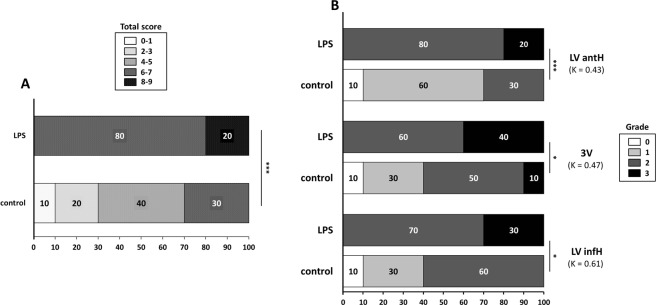
Scoring of *in-vivo* MR signal drops in ChPs. Percentage distribution of the total scores in LPS-treated and control mice (**A**). Percentage distribution of the individual grades for each ChP location (LV antH, 3 V, and LV infH ChPs) in LPS-treated and control mice (**B**). Numbers in parentheses indicate the Ƙ coefficient for inter-operator agreement (3 operators). Significant differences between LPS-treated group and control group, calculated with a Cochran Armitage test, are indicated by *for p < 0.05; **for p < 0.01 and ***for p < 0.005. LV antH: lateral ventricle anterior horns; 3 V: third ventricle; LV infH: lateral ventricle inferior horns.

Next, to test the hypothesis that MR signal drops in ChPs allow to discriminate between control and LPS-treated mice, the signal drops on post-USPIOs MR images were blindly scored in each ChP location (LV antH, 3 V, LV infH and 4 V ChPs, Suppl. Fig. 1) by three independent operators (F.C., C.D., V.H.) using a 4-point scale, where grade 3 means marked, grade 2 moderate, grade 1 slight, and grade 0 no signal drop (Suppl. Fig. S4). There was a good agreement in scores between the 3 operators for the LV infH ChPs (κ = 0.61), a fair agreement for the 3 V ChPs (κ = 0.47) and the LV antH ChPs (κ = 0.43) (Fig. 3B) and a poor agreement for the 4 V ChPs (κ = 0.04). This was probably due to the fact that MR signal drops were heterogeneous from one slice to another in the 4 V (Suppl. Fig. S5), which resulted in a heterogeneous way of rating this location between the 3 operators.

To statistically investigate the differences between LPS-treated and control mice, we attributed a consensual score to each location based on the majority of the 3 operators’ scores. A total score was calculated by adding the consensual grades of the different ChP locations. Due to the poor agreement obtained in the 4 V ChPs, we decided to exclude this region from the total score analysis. The total score was thus calculated in the 3 remaining regions (LV antH, LV infH and 3 V), resulting in a 9-point score.

All LPS-treated mice had a total score >6 (Fig. 3A), including those treated with 2.5 and 10 mg LPS/kg. Therefore all animals that had received 2 mmol Fe/kg were pooled in the statistical analysis, resulting in a total of 20 included mice (10 control mice and 10 LPS-treated mice). The results showed statistically significant higher scores in the LPS-treated group (Fig. 3A). This was true for each individual ChP location (Fig. 3B), including the 4 V (p = 0.045). When considering the threshold of 6 for the total score to discriminate mice with neuroinflammation from mice without, the method had negative predictive value of 100% and positive predictive value of 77%.

In addition, the 3 operators blindly and independently attributed a score going from 0 to 2 to reflect the presence of hypointense spots inside the mouse brain parenchyma (absence = 0; moderate = 1; important = 2). There was a fair agreement between the scores of the 3 operators (κ = 0.56). When looking at the consensual scores, no spots were present in control mice while 80% of LPS-treated mice had intracerebral hypointense spots (Suppl. Fig. S6A), resulting in a negative predictive value of 83% and a positive predictive value of 100%.

**Figure 4:**
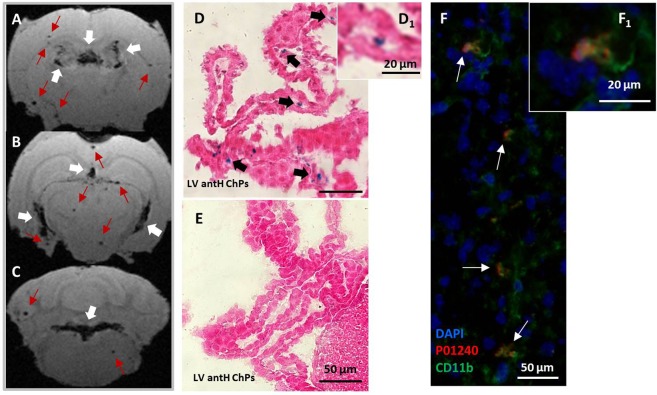
*Ex-vivo* detection of MR signal drops and of USPIOs presence in ChPs. *Ex-vivo* T2*-weighted images of a representative 5 mg/kg LPS-treated mouse injected with P904 at the dose of 2 mmol Fe/kg (**A–C**). Only one slice is shown per ChPs location. White arrows indicate signal drops inside the ChPs of the LV antH and 3 V (**A**), the LV infH and 3 V (**B**) and the 4 V (**C**), while red arrows show hypointense spots inside the mouse brain parenchyma. The MR signal drops detected *in-vivo* persisted *ex-vivo* after washing out of the vascular space. Histological brain sections stained with Prussian blue (PB) to detect P904-USPIOs (**D,E**). PB-spots were visualized inside the ChP stroma of LPS-treated mice (black arrows in D and magnification D_1_), but not in the ChPs of control mice (**E**). Brain immunofluorescence section of the LV intH ChPs in a representative LPS-treated mouse injected with P01240 (blue: DAPI; red: P01240; Green: CD11b). There was a colocalization between fluorescent P904 (P01240) and CD11b positive cells (white arrows in F and magnification F_1_).

To determine the exact location of USPIOs inside ChPs, we performed *ex-vivo* high resolution MRI and histological analysis. As ChPs are highly vascularized organs, we wanted to make sure that MR signal drops did not come from P904 trapping within ChP capillaries^24^. Mice were thus euthanized by intracardiac perfusion in order to remove any USPIO that could remain within the blood sector. On *ex-vivo* high resolution T2*-weighted MRI (59-µm in-plane and 160 µm slice thickness, 10 hours acquisition time), MR signal drops were still observed in all ChPs of LPS-treated mice (Fig. 4A–C, white arrows and Supplementary Video). Hypointense spots were also still detected inside the brain parenchyma (red arrows, Fig. 4A–C). The next step was to evaluate the presence of USPIOs inside the ChPs using Prussian blue (PB) iron staining. We found an important number of PB blue spots in all ChPs of LPS-treated mice (Fig. 4D). PB blue spots are located within the ChP stroma, between ChP capillaries and ChP epithelium (black arrows on Fig. 4D, and Figure 4D_1_) and did not seem to be associated with either endothelial or epithelial cells. In addition, PB staining was seen in the brain parenchyma in colocalization with hypointense spots seen on T2*-weighted imaging (Suppl. Fig. S6B). As shown in Fig. 4E, PB blue spots were not detected in control mouse ChPs. To investigate whether P904-USPIOs had been internalized by ChP immune cells, some of the mice had been injected with fluorescent P904 (P01240: P904 coupled with rhodamine). CD11b immunostaining was used as a marker of myeloid cells. In LPS-treated mice, there was visually more CD11b+ cells in the ChPs compared to controls. In addition, CD11b+ cells were found spread out in the brain parenchyma in this group only (Suppl. Fig. S6C). P904 fluorescent signal (Fig. 4F) matched the area of signal drop in MRI (Fig. 2A, LV infH, see the right white arrow). P904 fluorescent signals were observed within the ChP stroma and systematically colocalized with CD11b-positive immune cells (white arrows Fig. 4F, focused on the LV infH ChPs). This was most likely due to internalization of the contrast agent by myeloid cells (Figure 4F_1_). In contrast, we did not detect any P01240 red spots in the ChPs of control mice.

**Figure 5:**
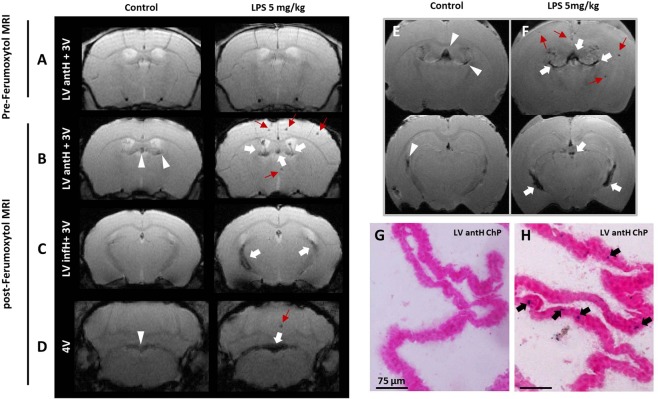
Replication of results using the FDA-approved USPIO Ferumoxytol. Pre-USPIO (**A**) and post-USPIO (**B–D**) T2*-weighted images of two representative mice: a control mouse and a mouse treated with 5 mg/kg of LPS, both injected with Ferumoxytol at the dose of 2 mmol Fe/kg. Only one slice is shown per ChP location and the pre-USPIO image is shown for two ChPs locations only (LV antH and 3 V). *Ex vivo* T2*-weighted images for a control mouse (**E**) and a mouse injected with 5 mg/kg of LPS (**F**). White arrows indicate signal drops in the ChPs of the LPS-treated mice. Red arrows show hypointense spots in the brain parenchyma of the LPS-treated mice. White arrowheads indicate slight hypointense signals inside the ChPs of the control mice. Only 2 ChPs locations are shown and only one slice per location is shown. Prussian Blue staining performed on brain histological sections shows an accumulation of Ferumoxytol in the ChPs of an LPS-treated mouse (**G**, black arrows) but not in the ChPs of a control mouse (**H**).

Finally, to evaluate the translational potential for this USPIO-enhanced MRI approach, we replicated the study using Ferumoxytol (n = 6). The same marked signal drops were seen 48 h post-USPIOs injection in the ChPs of LPS-treated mice (Fig. 5A–D). As for P904, this effect was lost when the iron dose was reduced to 0.45 or 1 mmol Fe/kg (Suppl. Fig. S7). The hypointense signals were still detectable after blood sector wash-out by intracardiac perfusion on *ex-vivo* high resolution MRI with slight signal drops in control mice and marked signal drops in LPS-treated mice (Fig. 5E,F). On brain histological sections, PB-blue spots signals were observed within the choroid plexus stroma as with P904 (Fig. 5H).

## Discussion

In this study we used high resolution MRI to reveal inflammatory alterations at the ChPs in a mouse model of neuroinflammation. Using *in-vivo* MRI, we identified extensive USPIO-related signal drops in the ChPs of animals with neuroinflammation compared to controls. *Ex-vivo* analysis, including MRI and immunohistology, confirmed USPIOs accumulation within the ChP stroma and their uptake by myeloid cells. We validated the translational potential of our approach using the FDA-approved iron oxide Ferumoxytol. Altogether these results suggest that minimally invasive MR imaging of USPIOs accumulation within the ChPs may serve as an imaging biomarker of ChPs involvement in presence of neuroinflammation.

Two distinct and probably complementary mechanisms may explain USPIOs accumulation within the ChPs of mice with neuroinflammation. The first mechanism is the so-called “Trojan horse mechanism” by which activated peripheral immune cells may internalize USPIOs in the blood stream before being subsequently recruited at the ChPs by inflammatory signals. The second mechanism is represented by the crossing of ChP endothelium by free USPIOs, whether actively or passively, followed by secondary internalization by phagocytic cells present in the ChP stroma^26^. We have chosen to image the mouse brains 48 h after USPIOs injection for two reasons: first, to ensure that they have been eliminated from the blood circulation at the time of imaging (the plasma half-life of a 2-mmol Fe/kg dose of P904 in mice is 3.5 h^27^) and second, to allow time for the internalization of free USPIOs by phagocytic cells (whether circulating or ChP resident). There was a colocalization between CD11b positive cells and fluorescent USPIOs within the ChPs, thus suggesting that at this time-point, MRI signal drops reflect at least in part phagocytic activity at the ChPs. This result is in line with the findings of Millward and coll who demonstrated that many fluorescent VSOPs colocalized with ChP macrophages in EAE^15^. In the current study, the CD11b+/USPIO+ cells are also most probably macrophages, originating either from the activation of the innate immune sentinel cells that inhabit the ChPs^28^ or from infiltrating monocytes that are attracted to the ChPs by inflammatory signals. The other immune cells that have the potential to internalize USPIOs are neutrophils^19^, which are also CD11b+ myeloid cells. A potential limitation of our approach is that it does not allow to discriminate stromal-resident from infiltrated phagocytic cells. Nonetheless, regardless of their origin, the stronger presence of phagocytic cells within the ChPs of LPS-treated mice compared to control mice is an indicator of a neuroinflammatory state. In relation to ChPs, endothelial and epithelial cells have been shown to internalize VSOP to a lesser extent in EAE^14,15^. It did not seem to be the case in our study, probably because nanoparticles with different properties (and size in particular) get differently internalized.

Surprisingly, slight to moderate MRI signal drops were also detected in the ChPs of control mice. Contrary to the blood brain barrier capillaries, ChP capillaries are fenestrated. In the healthy ChPs, the pore size is around 12 nm^29^, which is much lower than the USPIOs hydrodynamic diameter (25–30 nm). Thus we did not expect a passive diffusion of USPIOs through the ChP capillary fenestrations (at least not in control animals). Importantly, these findings were not matched by PB staining on histology. This argues in favor of a stromal localization of free USPIOs in control animals, as PB staining technique is known to lack sensitivity for the detection of interstitial USPIOs (as opposed to cell-internalized USPIOs)^30^. Altogether, these findings may indicate an active transport of USPIOs through the ChP endothelium by transcytosis. Endothelium transcytosis of bare iron oxide nanoparticles is known to occur slowly and in small proportion in the brain, even under physiological conditions^31,32^. This phenomenon is both concentration- and time-dependent^32^, which might explain why USPIOs accumulation was only observed using a relatively high dose of iron that provides both elevated plasma concentration and prolonged circulation times. Of note, the dose of 2-mmol Fe/kg has been repeatedly used for the MR imaging of phagocytic cells in a mouse model of ischemic stroke without overt toxic effects^23–25^. The exact mechanisms by which USPIOs enter the ChP stroma warrants further investigations. Future studies should aim at: (i) determining the optimal USPIO dose to minimize hypointense signals in the healthy ChPs; (ii) optimizing the MR sequences in order to specifically detect USPIO-labelled cells^33,34^; and (iii) evaluate the fate of USPIOs at later time-points in the different groups.

In any case, the accumulation of USPIOs within ChPs was far more important in mice with neuroinflammation than in mice without, as seen visually on MRI and objectified by the significant differences in scores between the two groups. This is going along the lines of an internalization of USPIOs inside phagocytic cells, as USPIO-laden cells are known to induce stronger MR signal drops than free USPIOs at equal iron concentration^34^. In addition, there seems to be differences in USPIO accumulation according to ChPs location, with the ChPs residing in the anterior horn of the lateral ventricles being the most discriminant between the two groups. There were also some heterogeneities within each ChP location which may have somewhat compromised inter-observer agreements. Again, this had different impacts according to ChPs location, the 4 V showing the most heterogeneities and hence being the most difficult to synthesize into a single score. In addition to ChPs signal drops, hypointense spots were observed in the brain parenchyma of mice with neuroinflammation. This phenomenon has already been described using a protocol similar to ours and is thought to reflect the transmigration of peripheral phagocytes into mouse brains^35^. Because of non-specific iron oxide uptake into the ChPs of healthy mice, a substantial proportion of animals not subjected to neuroinflammation had non-negligible ChPs signal drops (30%). The probability that the presence of signal drops in ChPs was related to neuroinflammation (positive predictive value) was thus 77%. In addition, the probability that the presence of hypointense spots in the brain parenchyma was related to neuroinflammation was 100%. Hence, if this method was to be used to monitor animals with a chronic neuroinflammatory disease, the combination of the two radiological signs on USPIO-enhanced MRI (ChP signal drops + hypointense spots) may help stratify the animals with ChPs signal drops that actually present neuroinflammation, with a positive predictive value of 100%.

The development of an *in-vivo* imaging tool allowing to study the involvement of ChPs in neuroinflammatory disorders is crucial for better understanding the physiopathology of these diseases and for monitoring the effects of immunomodulatory interventions. We show in the present study that USPIO-enhanced MRI has the potential to fulfil this aim not only at the pre-clinical level but also at the clinical level, as our results were obtained using a clinically-applicable contrast agent. However, there are several limitations to the present setting that should be addressed in future works. First, we have chosen to induce neuroinflammation by intraperitoneal administration of LPS, because it is a simple and well-characterized model^22^. A single i.p. LPS injection at 5 mg/kg results in the production of TNFα and other pro-inflammatory cytokines in the periphery^36^. The entry of these pro-inflammatory mediators into the brain causes the rapid (first hours to days) activation of microglia that continues for weeks to months after the initial stimulus has ceased, resulting in a persistent and self-propelling neuroinflammation^22^. Further evaluation in models of human diseases with a neuroinflammatory component, such as multiple sclerosis, glioma or stroke, should be performed to validate the approach, both retrospectively and prospectively. Second, this study evaluated only a single time-point post-USPIOs. It would be of interest to investigate the possibility for longitudinal monitoring and/or to determine whether this approach could provide a marker of disease severity, natural evolution, or regression upon treatment. For example, Kirschbaum and coll found that there was a correlation between Ferumoxytol uptake in the brain parenchyma and clinical disease severity in EAE^19^ while Marinescu *et al*. showed that USPIO-enhanced MRI allowed to monitor the anti-inflammatory effects of minocycline in the brain of mice with ischemic stroke^25^. In EAE, Millward and coll showed that VSOPs were absent from the ChPs during remission, but accumulated again during subsequent relapse^14,15^. In contrast, we did not detect any significant difference in signal drops when the LPS dose was increased. Further investigations need to be done to determine if this is due to a lack of sensitivity of the approach, or to the fact that LPS dose increase does not induce an increase in activated immune cells within the ChPs.

In summary, we have shown that USPIO-enhanced MRI may be applied to study ChPs involvement in neuroinflammatory disorders. USPIO-enhanced MRI may prove useful to study neuroimmunological interfaces *in-vivo*. Although there are still several limitations to overcome (such as elucidating the route of entrance of UPSIOs into the ChPs, defining the optimal USPIOs dose and MR sequences and evaluating the long-term fate of USPIOs), this approach is attractive as it could be applied in a straightforward way in clinical practice.

## Methods

### Animal experiments

All experimental procedures involving animals and their care were carried out in accordance with the European regulation for animal use (EEC Council Directive 2010/63/UE, OJ L 276, Oct. 20, 2010) and this study was approved by our local ethic committee “Comité d’éthique pour l’Expérimentation Animale Neurosciences Lyon” (CELYNE - CNREEA number: C2EA – 42). The animals were housed in a temperature and humidity-controlled environment (21.2 ± 3 °C), on 12:12 h light-dark cycle, having free access to standard chow and tap water. All animal experiments were performed in 8-week-old (25.1+/−2.7 g) C57Bl/6 male mice (*n* = 31, Janvier, France). The animal model of neuroinflammation consisted in an intraperitoneal (ip injection (150 µl) of lipopolysaccharide (LPS) from *Escherichia coli* 0111:B4 (dissolved in saline; cat: L-2630, Sigma-Aldrich, Saint-Louis, USA).

### Contrast media for MRI

Two kinds of ultrasmall super paramagnetic particles of iron oxide (USPIO) were used as MRI contrast agent for this study: P904 USPIO (Chematech, Guerbet, Dijon, France) and its fluorescent version (P01240: P904 labelled with rhodamine, Chematech, Guerbet, Dijon, France) and an FDA-approved USPIO, Ferumoxytol (AMAG pharmaceutical, Waltham, USA).

P904 consists of pegylated particles with a hydrodynamic diameter of 25 to 30 nm and are supplied as 450 µl solutions with an iron concentration of 80 mmol/L. Ferumoxytol consists of carboxymethyl dextran-coated particles with a hydrodynamic diameter of 17 to 31 nm and they are supplied as 17 ml solutions with an iron concentration of 536 mmol/kg.

USPIOs were dissolved in saline and injected intravenously into the retro-orbital vein as a final volume of 150 µl.

### MRI procedures

For *in-vivo* MRI, mouse anesthesia was induced with a mixture of air and 3.5% isoflurane (ISO-VET, Piramal Healthcare, Morpeth, UK) and then animals were placed in an MRI-compatible mouse cradle. During the acquisitions, anesthesia was maintained at 2% isoflurane. The respiratory rhythm was cautiously monitored by a pressure sensor linked to a monitoring system (ECG Trigger Unit HR V2.0, RAPID Biomedical, Rimpar, Germany), as well as the body temperature thanks to circulating heated water. MRI was performed on a 7 T horizontal-bore Bruker Avance II rodent imaging system (Bruker Biospin, Ettlingen, Germany), using a 50-mm inner diameter birdcage coil for transmission and a 15-mm diameter surface coil for reception. For each sequence, 25 slices were acquired from the olfactory bulb to the cerebellum, using a field of view (FOV) of 20 × 20 mm^2^, a slice thickness of 500 µm and a matrix size of 256 × 256. The *in-vivo* MRI protocol comprised the following axial sequences: a spin-echo T2 weighted image (T2-WI), TE/TR = 43.8/5000 ms, bandwidth = 40 kHz, number of averages = 6, acquisition time 12 min; a T2-star gradient echo FLASH sequence (T2*-WI), TE/TR = 6/750 ms, bandwidth = 40 kHz, flip angle (FA) = 20°, number of averages = 8, acquisition time 19 min; These parameters yielded an in-plane resolution of 78 µm.

For *ex-vivo* scans, mice were euthanized by intracardiac perfusion with phosphate-buffered saline (PBS) followed by fixation with 4% paraformaldehyde (PFA) dissolved in PBS. Brains were then extracted and placed inside 2 ml syringes to maintain them in a static position. Syringes were filled with proton-free solvent Fluorinert (3 M™ Fluorinert™ Electronic Liquids, 3 M, St Paul, USA). A FLASH T2*-WI was acquired on the 7 T horizontal-bore Bruker Avance II system, with the following experimental parameters: TE/TR = 8/75 ms, bandwidth = 25 kHz, FA = 20°, number of averages = 14, slice thickness = 160 µm, FOV = 12.5 × 12.5 mm^2^ and matrix size = 256 × 256, acquisition time 10 hours. These parameters yielded an in-plane resolution of 59 µm.

### MRI signal and statistical analysis

Image analysis was performed using ImageJ software (National Institute of Health, USA imagej.nih.gox/ij/). The MR signal drop in ChPs was assessed using a scoring system. This score was attributed by three independent blind operators. Four regions were investigated (Suppl. Fig. 1): ChPs of left and right lateral ventricle anterior horns (LV antH ChP, extended on three MRI slices), ChPs of the third ventricle (3 V ChP, extended on three MRI slices), ChPs of left and right lateral ventricle body and inferior horns (LV infH ChP, extended on two MRI slices) and ChPs of the fourth ventricle (4 V ChP, extended on three MRI slices). For each region, a grade going from 0 to 3 was given by each operator, according to the signal drop intensity within ChPs (Suppl. Fig. 4). A kappa de fleiss analysis was performed to evaluate the level of agreement between the 3 operators with respect to the degree of contrast drop. It consisted in calculating the κ coefficient for inter-operator agreement (κ < 0.20 = poor, κ 0.21–0.40 = fair, κ 0.41–0.60 = moderate, κ 0.61–0.80 = good, κ 0.81–0.90 = very good, κ > 0.90 = excellent agreement). A consensual score was given based on the majority of the 3 operators’ scores. The total score was obtained by adding the consensual scores. In addition, a grade going from 0 to 2 was given for the presence or not of hypointense spots within brain parenchyma. To determine differences in ChP signal drop between control and LPS-treated mice, a Cochran Armitage test (R statistical software version 3.3.3 (2017–03–06) “Another Canoe.”) was used. In all comparisons, a p-value < 0.05 was considered statistically significant.

### Histology and immunofluorescence

For histological analysis, mice were euthanized by intracardiac perfusion with PBS. Brains were then removed and frozen in methylbutane with dry ice. Finally, tissues were cut into 12 µm sections on a cryostat. In a first set of experiment, Prussian blue staining was done for iron detection using Perl’s method (Iron Stain kit, Sigma-Aldrich, Saint-Louis, USA): slides were incubated with 1% hydrochloric acid and 1% potassium hexacyanoferrate for one hour. Hematoxylin and eosin (H&E) staining was used for the counterstaining of nucleus and cytoplasm. The slides were then mounted with Roti-Mount® Fluocare with DAPI (Roth, Lauterbourg, France). In a second set of experiment (mice injected with fluorescent P904), CD11b immunofluorescence was performed for detection of CD11b positive cells. Briefly, slides were rinsed three times with PBS. They were then blocked and permeabilized in a solution of 5% Bovine Serum Albumin (BSA, Sigma-Aldrich, Saint Louis, USA) and 0.5% triton (Sigma-Aldrich, Saint Louis, USA) for 30 minutes at room temperature. Slides were then rinsed with 0.5% PBS-Triton (PBST) and incubated with primary antibody for CD11b (1:500; MCA275G, AbD Serotec, BioRad, Hercules, USA) diluted in PBST, overnight at 4 °C. They were then washed three times in PBS and incubated with a secondary anti-rat antibody (1:1000, A-21471, A594 chicken anti-rat, ThermoFisher, Waltham, USA). Finally, slides were rinsed three times in PBST, then mounted with Roti-Mount® Fluocare with DAPI and investigated for the presence of fluorescent P904 and CD11b positive cells. Images were acquired from the 12 µm section using an Axio Scope A.1 fluorescence microscope (4 filters, Carl Zeiss, Oberkochen, Germany) equipped with a x0.63 AxioCam MRc (Carl Zeiss, Oberkochen, Germany).

## Supplementary information

Dataset 1

Supplementary video

## Data Availability

The datasets generated during and/or analyzed during the current study are available from the corresponding author on reasonable request.
