# Supplementary material for: MRI coupled with clinically-applicable iron oxide nanoparticles reveals choroid plexus involvement in a murine model of neuroinflammation
Source: Sci Rep. 2019 Jul 11;9:10046. doi: 10.1038/s41598-019-46566-1 (PMC6624288; doi:10.1038/s41598-019-46566-1)
Supplement: Supplementary file 1 — Dataset 1 [file 41598_2019_46566_MOESM1_ESM.docx]

***Supplementary information***

**MRI coupled with clinically-applicable iron oxide nanoparticles** **reveals choroid plexus involvement in a murine model of neuroinflammation**

**Violaine Hubert, Chloé Dumot, Elodie Ong, Camille Amaz,**

**Emmanuelle Canet-Soulas, Fabien Chauveau_,_ Marlène Wiart***

*** Correspondence:** Corresponding Author: [marlene.wiart@univ-lyon1.fr](mailto:marlene.wiart@univ-lyon1.fr)

**Supplementary Figure S1 │ Visualization of ChPs anatomical location on MRI.** Visualization of all ChPs location (black arrows) on baseline T2-weighted images: ChPs of lateral ventricle anterior horns (LV antH); ChPs of the third ventricle (3V); ChPs of lateral ventricle inferior horns (LV infH) and ChPs of the fourth ventricle (4V).

**Supplementary Figure S2 │ P904 dose-response trial in LPS-treated mice.** Post-USPIO T2*-weighted images of representative mice treated with 5 mg/kg LPS and injected with P904 at different doses (A). The marked signal drops that appeared in ChPs with 2 mmol Fe/kg (A_1_, white arrows) are no longer present at 1 mmol Fe/kg (A_2_) and at 0.45 mmol Fe/kg (A_3_). Only one slice is shown per ChPs location. Prussian blue coloration (PB) on histological brain sections of mice treated with 5 mg/kg LPS (B). Several PB-spots were detected inside the ChPs of mice injected with 2 mmol Fe/kg of P904 (B_1_, black arrows) but not with 1 mmol Fe/kg (B_2_) nor with 0.45 mmol Fe/kg (B_3_).

**Supplementary Figure S3 │ Impact of LPS dose on MR signal drops in ChPs.** Pre-USPIO (A) and post-USPIO (B-D) T2*-weighted images of 4 representative mice: a control mouse, a mouse treated with 2.5 mg/kg of LPS, a mouse treated with 5 mg/kg of LPS and a mouse treated with 10 mg/kg of LPS, all injected with P904 at the dose of 2 mmol Fe/kg. White arrows indicate signal drops inside the ChPs of the LPS-treated mouse. Red arrows show hypointense spots inside the brain parenchyma of LPS-treated mice. White arrowheads indicate slight signal drops inside the ChPs of the control mouse. There was no obvious LPS dose effect on the intensity of MR signal drops. Only one slice is shown per ChP location and the pre-USPIO image is shown for two ChP locations only (LV antH and 3V). LV antH: lateral ventricle anterior horns; 3V: third ventricle; LV infH: lateral ventricle inferior horns; 4V: fourth ventricle.

**Supplementary Figure S4 │ Illustration of the scoring system.** Post-USPIO T2*-weighted images of representative mice injected with P904 at the dose of 2 mmol Fe/kg, rated as grade 0 (no signal drop), grade 1 (slight signal drop), grade 2 (moderate signal drop) and grade 3 (marked signal drop) for each ChP location. Only one slice is shown per ChPs location but the operators rated the animals based on all slices.

**Supplementary Figure S5 │ Signal drop heterogeneity within the 4V ChPs**. Post-USPIO T2*-weighted images of a single LPS-treated mouse injected with P904 at the dose of 2 mmol Fe/kg showing that MR signal drops in the 4V ChPs are heterogeneous from one slice to another (black arrows) and hence difficult to score.

**Supplementary Figure S6 │ Supplementary Figure S6 │ Presence of hypointense spots and CD11b+ cells within the brain parenchyma of LPS-treated mice.** Percentage distribution of the 3-point score reflecting the presence of hypointense spots in the brain parenchyma, for LPS-treated and control mice (A). Numbers in parentheses indicate the k coefficient for inter-operator agreement (3 operators). Significant differences between LPS-treated group and control group, calculated with a Cochran Armitage test, are indicated by *** for p <0.005. An example of Prussian blue coloration (PB) on a histological brain section of a 5 mg/kg LPS-treated mouse (B) revealed the presence of several PB-spots within the brain parenchyma (B_2_), which matched the location of hypointense spots observed on T2*-weighted images (B_1_). CD11b immunolabelling (C) shows the presence of CD11b+ cells in the brain parenchyma of LPS-treated mice (C_1_, white arrow heads) but not of control mice (C_2_). Blue: DAPI; Green: CD11b.

**Supplementary Figure S7 │ Ferumoxytol dose-response trial in LPS-treated mice.** Post-USPIO T2*-weighted images of representative mice treated with 5 mg/kg LPS and injected with Ferumoxytol at different doses (A). The marked signal drops that appeared in ChPs with 2 mmol Fe/kg (A_1_, white arrows) are no longer present at 1 mmol Fe/kg (A_2_) and at 0.45 mmol Fe/kg (A_3_). Only one slice is shown per ChPs location. Prussian blue coloration (PB) on histological brain sections of mice treated with 5 mg/kg LPS (B). Several PB-spots were detected inside the ChPs of mice injected with 2 mmol Fe/kg of Ferumoxytol (B_1_, black arrows) but not with 1 mmol Fe/kg (B_2_) nor with 0.45 mmol Fe/kg (B_3_).

**Supplementary Video │ Ex-vivo high resolution T2* weighted images of a representative mouse injected with 5 mg/kg of LPS.**

(Video uploaded in a separate file)

**Supplementary Figures**


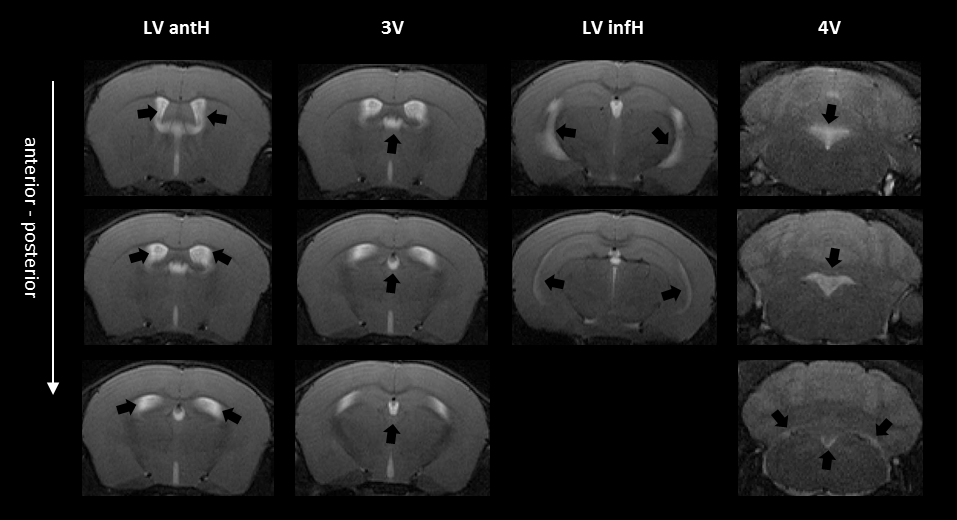


**Supplementary Figure S1 │ Visualization of ChPs anatomical location on MRI.** Visualization of all ChPs location (black arrows) on baseline T2-weighted images: ChPs of lateral ventricle anterior horns (LV antH); ChPs of the third ventricle (3V); ChPs of lateral ventricle inferior horns (LV infH) and ChPs of the fourth ventricle (4V).


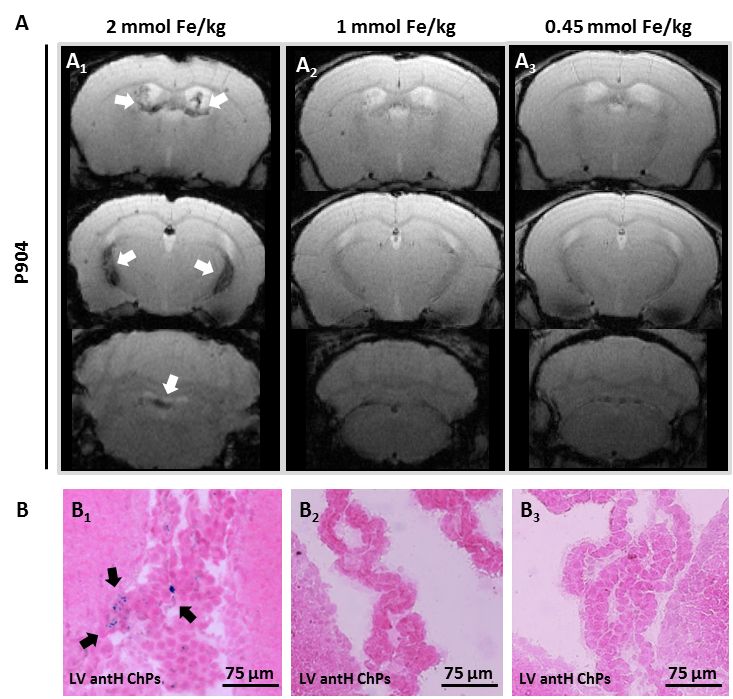


**Supplementary Figure S2 │ P904 dose-response trial in LPS-treated mice.** Post-USPIO T2*-weighted images of representative mice treated with 5 mg/kg LPS and injected with P904 at different doses (A). The marked signal drops that appeared in ChPs with 2 mmol Fe/kg (A_1_, white arrows) are no longer present at 1 mmol Fe/kg (A_2_) and at 0.45 mmol Fe/kg (A_3_). Only one slice is shown per ChPs location. Prussian blue coloration (PB) on histological brain sections of mice treated with 5 mg/kg LPS (B). Several PB-spots were detected inside the ChPs of mice injected with 2 mmol Fe/kg of P904 (B_1_, black arrows) but not with 1 mmol Fe/kg (B_2_) nor with 0.45 mmol Fe/kg (B_3_).


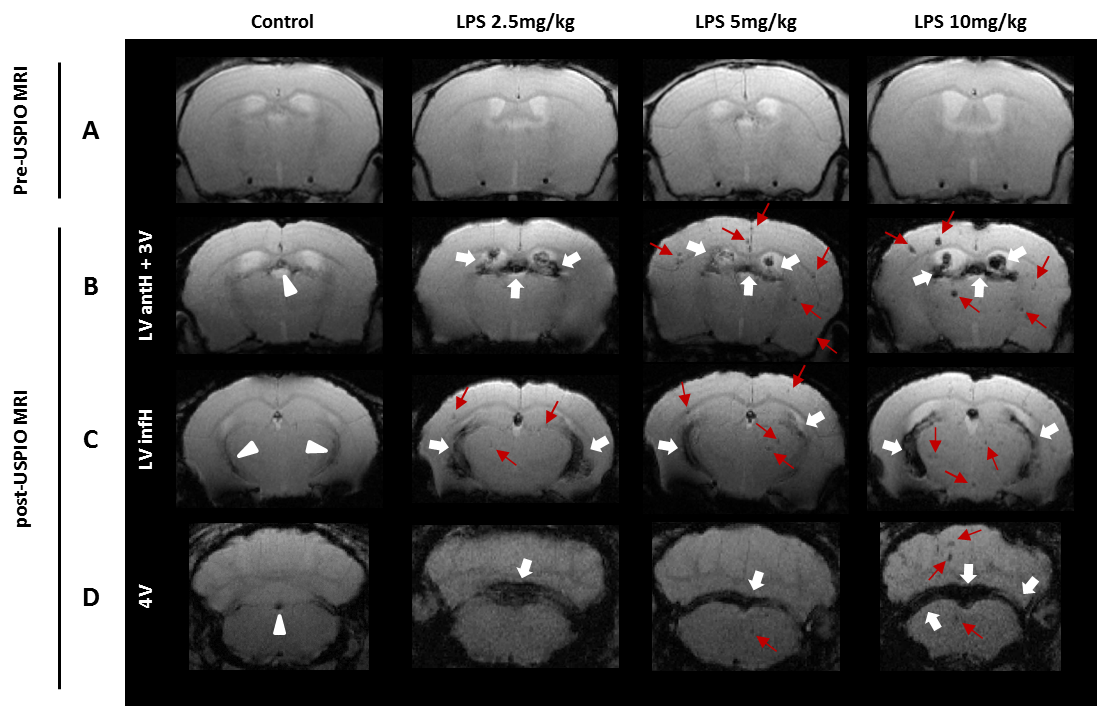


**Supplementary Figure S3 │ Impact of LPS dose on MR signal drops in ChPs.** Pre-USPIO (A) and post-USPIO (B-D) T2*-weighted images of 4 representative mice: a control mouse, a mouse treated with 2.5 mg/kg of LPS, a mouse treated with 5 mg/kg of LPS and a mouse treated with 10 mg/kg of LPS, all injected with P904 at the dose of 2 mmol Fe/kg. White arrows indicate signal drops inside the ChPs of the LPS-treated mouse. Red arrows show hypointense spots inside the brain parenchyma of LPS-treated mice. White arrowheads indicate slight signal drops inside the ChPs of the control mouse. There was no obvious LPS dose effect on the intensity of MR signal drops. Only one slice is shown per ChP location and the pre-USPIO image is shown for two ChP locations only (LV antH and 3V). LV antH: lateral ventricle anterior horns; 3V: third ventricle; LV infH: lateral ventricle inferior horns; 4V: fourth ventricle.


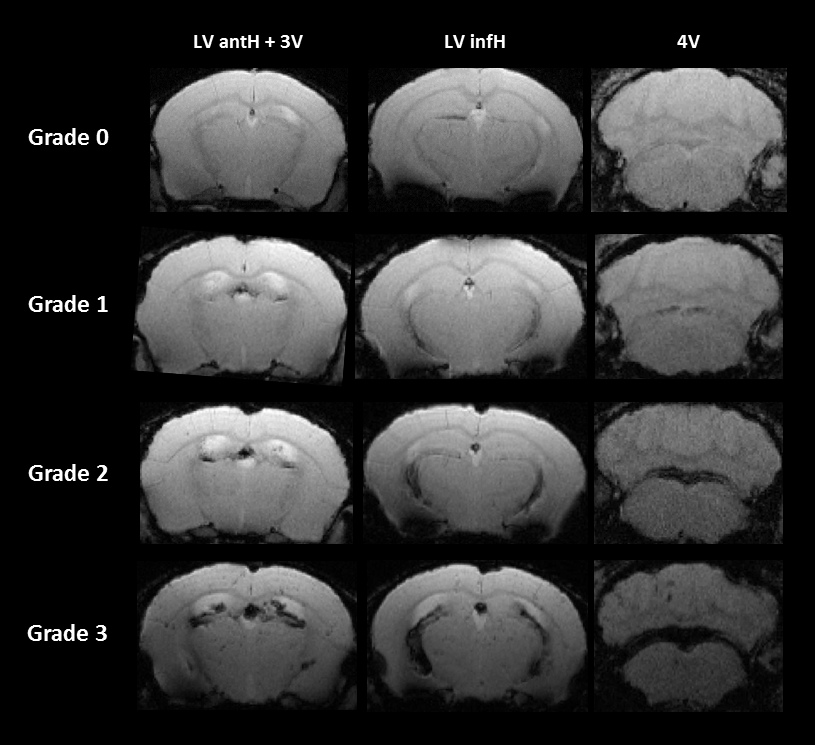


**Supplementary Figure S4 │ Illustration of the scoring system.** Post-USPIO T2*-weighted images of representative mice injected with P904 at the dose of 2 mmol Fe/kg, rated as grade 0 (no signal drop), grade 1 (slight signal drop), grade 2 (moderate signal drop) and grade 3 (marked signal drop) for each ChP location. Only one slice is shown per ChPs location but the operators rated the animals based on all slices.


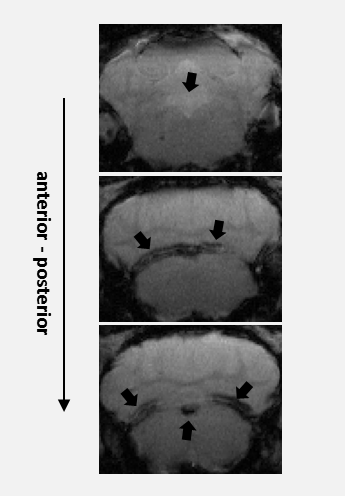


**Supplementary Figure S5 │ Signal drop heterogeneity within the 4V ChPs.** Post-USPIO T2*-weighted images of a single LPS-treated mouse injected with P904 at the dose of 2 mmol Fe/kg showing that MR signal drops in the 4V ChPs are heterogeneous from one slice to another (black arrows) and hence difficult to score.


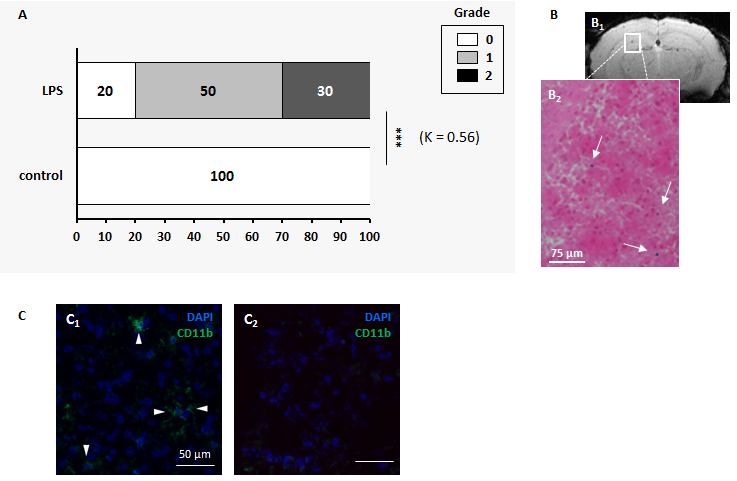


**Supplementary Figure S6 │ Presence of hypointense spots and CD11b+ cells within the brain parenchyma of LPS-treated mice.** Percentage distribution of the 3-point score reflecting the presence of hypointense spots in the brain parenchyma, for LPS-treated and control mice (A). Numbers in parentheses indicate the k coefficient for inter-operator agreement (3 operators). Significant differences between LPS-treated group and control group, calculated with a Cochran Armitage test, are indicated by *** for p <0.005. An example of Prussian blue coloration (PB) on a histological brain section of a 5 mg/kg LPS-treated mouse (B) revealed the presence of several PB-spots within the brain parenchyma (B_2_), which matched the location of hypointense spots observed on T2*-weighted images (B_1_). CD11b immunolabelling (C) shows the presence of CD11b+ cells in the brain parenchyma of LPS-treated mice (C_1_, white arrow heads) but not of control mice (C_2_). Blue: DAPI; Green: CD11b.


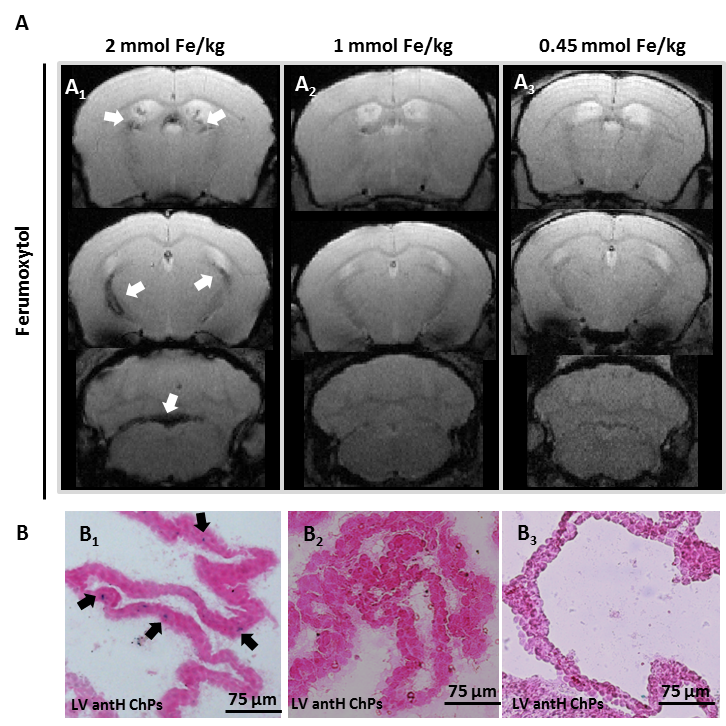


**Supplementary Figure S7 │ Ferumoxytol dose-response trial in LPS-treated mice.** Post-USPIO T2*-weighted images of representative mice treated with 5 mg/kg LPS and injected with Ferumoxytol at different doses (A). The marked signal drops that appeared in ChPs with 2 mmol Fe/kg (A_1_, white arrows) are no longer present at 1 mmol Fe/kg (A_2_) and at 0.45 mmol Fe/kg (A_3_). Only one slice is shown per ChPs location. Prussian blue coloration (PB) on histological brain sections of mice treated with 5 mg/kg LPS (B). Several PB-spots were detected inside the ChPs of mice injected with 2 mmol Fe/kg of Ferumoxytol (B_1_, black arrows) but not with 1 mmol Fe/kg (B_2_) nor with 0.45 mmol Fe/kg (B_3_).
